# Transcriptomic analysis of the TRP gene family in human brain physiopathology

**DOI:** 10.3389/fnmol.2025.1576941

**Published:** 2025-04-24

**Authors:** Barbara Olejniczak, Arpita Balakrishnan, Justyna Augustyniak, Elżbieta Salińska, Agnieszka Bronisz, Jakub Godlewski

**Affiliations:** ^1^Department of Neurooncology, Mossakowski Medical Research Institute, Polish Academy of Sciences, Warsaw, Poland; ^2^Translational Medicine Doctoral School, Centre of Postgraduate Medical Education, Warsaw, Poland; ^3^Tumor Microenvironment Laboratory, Mossakowski Medical Research Institute, Polish Academy of Sciences, Warsaw, Poland; ^4^Department of Neurochemistry, Mossakowski Medical Research Institute, Polish Academy of Sciences, Warsaw, Poland

**Keywords:** transient receptor potential (TRP), brain physiopathology, transcriptomic analysis, dementia, neuroscience

## Abstract

The transient receptor potential (TRP) gene family is vital to cellular physiology, mediating ion flow across membranes and facilitating sensory signal transduction. This article examines the transcriptomic landscape of TRP genes, emphasizing their varying expression across organs, tissues, and cells, with a particular focus on the brain. Analysis reveals a distinct spatial distribution of TRP gene expression, notably enriched in the hippocampus during brain development, highlighting their essential role in neuronal function. Utilizing datasets from the Human Protein Atlas, Allen Human Brain Atlas, and studies on aging and dementia, associations are identified between TRP gene expression and the development or pathophysiology of neural tissue, highlighting the therapeutic potential of TRP channels in addressing, e.g., sensory impairments and cognitive decline. These insights into the regulatory dynamics of TRP channels lay a foundation for developing targeted interventions for neurodegenerative disorders.

## 1 Introduction

The transient receptor potential (TRP) gene family encodes a diverse group of non-selective cation channels critical for cellular physiology by facilitating ion flow across membranes. These channels play a pivotal role in sensory perception, responding to a range of external stimuli. The large human TRP superfamily, comprising 27 genes, classified into six major subfamilies based on distinct genomic structures, reflects their extensive functional roles in maintaining cellular homeostasis (Pan et al., 2011). These large and complex multi-subunit protein gene products are integral to cell membranes and crucial for sensory signal transduction across developmental stages. TRP channels trigger intracellular calcium signaling cascades upon activation, leading to tailored cellular responses depending on the stimulus (Zhang et al., 2023).

TRPs, expressed widely across neuronal and non-neuronal tissues, mediate sensory modalities, including pain, temperature, taste, and pressure, and serve as sensors for osmotic potentials, mechanical stretch, and vibration. Notably enriched in the brain, certain TRP family members support essential neuronal and glial functions (Sawamura et al., 2017), and their dysregulation, disrupting intracellular calcium homeostasis and signaling, is linked to neurodegenerative disorders and memory consolidation and retrieval accompanying brain aging (Lee et al., 2021). Insights into TRP channels’ unique expression patterns and roles in the brain reveal significant therapeutic potential for addressing sensory dysfunctions and cognitive impairments. Single-cell transcriptomics has provided high-resolution mapping of TRP gene expression across diverse cell types in the human cortex, identifying consistent enrichment or depletion patterns across brain tissues (Hodge et al., 2019). Despite challenges in data interpretation, such as the need to account for variable cell proportions in disease contexts like dementia, the therapeutic promise of TRP channels as therapeutic targets remains a *bona fide* research focus.

The distinct expression profiles of TRP genes across various organs and cells indicate a unique spatial distribution, particularly in the brain. Their enrichment in the developing hippocampus underscores their influence on regulating brain neuronal and glial developmental and homeostatic functions. This distinctive allocation of TRP genes opens up new avenues for research, particularly in understanding their role in memory network formation and their potential link to the pathophysiology of dementia in aging brains.

## 2 Methodology

### 2.1 Overall approach

The research applies a structured methodological approach, utilizing a variety of datasets compiled from healthy brain material harvested from developmental stages and aging brain tissues. These include brain cells from both physiological and dementia conditions, ensuring the reliability and validity of the findings while offering a detailed understanding of TRP gene expression in the brain. These datasets depict genomic coherency and alterations in human cells, revealing significant variability in cell transcriptome and contextualized biological geography. The initial analysis involves differential co-expression analysis (DCA) using data from the transcriptome atlas study, analyzed through bulk organ/tissue sample processing and single-cell sequencing to identify regionally enriched gene sets.

1. Data Collection—Datasets were selected based on their relevance to brain transcriptomics and the availability of comprehensive biospecimen metadata.
  - Identify relevant transcriptome datasets.
  - Specify criteria for sample inclusion/exclusion.
  - Extract TRP gene expression profiles across different tissues and brain developmental stages.
2. Pre-processing.
  - Filter genes to focus on TRP channel family members.
  - Normalize gene expression data.
  - Handle missing values and outliers.
3. DCA.
  - Integration of biospecimen characters from different datasets.
  - Compare TRP gene expression across different organ tissues.
  - Analyze co-expression patterns in the adult brain, developing brain, and aging brain.
  - Identify statistically significant differences in dementia.

### 2.2 Differential co-expression analysis

#### 2.2.1 Data collection and preprocessing

Transcriptome datasets for TRP gene family members were obtained from publicly available brain-specific gene expression repositories. These datasets were curated to include high-quality RNA sequencing (RNA-seq) data from various brain tissues or cell types. Normalized expression values calculated using the TPM (transcripts per million) method were used to account for sequencing depth and gene length.

#### 2.2.2 Differential co-expression network construction

Differential co-expression networks were constructed using the co-expression relationships between TRP family genes. The expression data of these genes were selected from the normalized transcriptome dataset. A co-expression matrix was generated using pairwise Pearson correlation coefficients between the expression profiles of each gene pair across all samples.

Brain-specific modules were identified through module eigengene analysis, and their biological significance was explored by correlating module expression with clinical or tissue-specific data.

#### 2.2.3 Principal component analysis

Principal component analysis (PCA) was performed on the gene expression data of TRP family members to reduce the dimensionality of the dataset and visualize the variance within the samples. PCA was conducted using the ClustVis R package (Metsalu and Vilo, 2015), and the first two principal components were visualized to identify clustering patterns among the samples. PCA results were used to evaluate the overall structure of the data and determine whether the expression profiles of TRP genes segregated in a way that corresponded to specific brain compartments or physiological conditions.

#### 2.2.4 Volcano plot analysis

A volcano plot-based analysis was carried out to identify genes that showed statistical significance and significant fold changes in expression. Differentially expressed genes (DEGs) between brain-specific modules were assessed using a two-sample t-test. Using the Benjamini–Hochberg method to control the false discovery rate (FDR), *p*-values were adjusted for multiple tests. Genes with a |log2 fold change| greater than one and an FDR-adjusted *p*-value of less than 0.05 were considered significant. The results were visualized using a volcano plot, with significant genes plotted according to their fold change and adjusted *p*-value.

#### 2.2.5 Hierarchical clustering analysis

Hierarchical clustering analysis of TRP gene expression was performed using Morpheus’s versatile matrix visualization and analysis software.^1^ This analysis was conducted for each dataset using hierarchical clustering and a similarity matrix to assess the relationships between TRP gene expression patterns. The characteristic expression profiles of TRP genes across different brain structures and cell types were examined. Mean, log-transformed, and normalized expression values (greater than 0.01 RKPM) from the datasets were used to define TRP gene signatures based on transcript expression across various tissues and cell types. This process adhered to all relevant laws, regulations, and policies for protecting human subjects, ensuring ethical data collection and usage.

### 2.3 Functional and statistical analysis

GO and KEGG pathway analyses were conducted using ShinyGo v0.80 software (Ge et al., 2020) and WebGestalt (Liao et al., 2019). Hierarchical clustering trees summarize the significant correlation among pathways using databases listed in the figures’ legends. All statistical operations are performed with GraphPad Prism 9 software and Excel, considering significance with a false discovery rate (FDR <0.05) with corrected *q*-value <0.01 and correlation *r* value = ±0.5.

### 2.4 Dataset and biomaterial sample types

The analysis of TRP gene expression utilized multiple publicly available datasets to ensure comprehensive coverage of normal and pathological states of human tissues and cells (for links and relevant citations, see Supplementary Data Sheet 1).

- The Human Protein Atlas^2^ was accessed to evaluate TRP gene expression across various normal human tissues, including 44 distinct tissues such as the brain, heart, liver, kidney, and lungs. This database provides RNA and protein expression data from diverse donors to capture baseline expression patterns reflective of normal physiological conditions.
- The Human Tissue Protein RNA Atlas^3^ was utilized to further analyze the expression levels of TRP genes across 54 different normal human tissues. The sampling strategy emphasized a broad age and sex diversity among healthy individuals to reflect physiological variations in tissue-specific expression.
- The Human Cells Protein RNA Atlas^4^ was consulted to examine the cell-type-specific expression of TRP genes. This Atlas contains profiles from 22 distinct cell types derived from various human tissues, including neuronal, epithelial, and connective tissues.
- For the analysis of brain region-specific TRP gene expression, the Allen Brain Map^5^ provided extensive data, including samples from 10 different brain regions, encompassing a total of 1,300 samples, with demographic diversity in terms of age, sex, and disease status among donors.
- The Human Brain Atlas^6^ provided a dataset including 21 brain regions and 660 samples from healthy individuals and those with neurodegenerative conditions to analyze brain-specific TRP gene expression in different anatomic areas.
- The Allen Brain Cell Atlas^7^ focuses on single-cell RNA sequencing data from post-mortem human brain tissue. This atlas provides insights into the transcriptomic profiles of various brain cell types. The sampling strategy includes over 170,000 single-cell transcriptomes obtained from multiple brain regions, enabling a detailed exploration of gene expression dynamics within specific cell populations, including neurons and glial cells.
- The Developing Human Brain Atlas^8^ focused on samples collected at various developmental stages, capturing changes in TRP gene expression from 8 post-conception weeks to 40 years. This dataset provided expression data from 42 individuals across 16 specific brain regions.
- The Allen Brain Atlas^9^ was utilized for single-cell resolution. It encompasses over 170,000 single-cell transcriptomes derived from 5 brain regions, including the cortex and hippocampus, representing diverse cell types like neurons and glial cells.
- The Aging, Dementia, and Traumatic Brain Injury Study^10^ contributed data from 107 samples, including 50 from dementia patients and 57 from age-matched controls. The study focused on the hippocampus and cortical regions to explore age-related changes in TRP gene expression.
- The Seattle Alzheimer’s Disease Brain Cell Atlas^11^ provided insight into the expression of TRP genes in samples from 69 individuals with varying levels of Alzheimer’s pathology, facilitating the investigation of disease-specific changes in TRP expression across different brain regions.
- Lastly, the CZ CELLxGENE Discover Platform,^12^ developed by Chan Zuckerberg, pairs data and tools to find, download, explore, analyze, and publish standardized single-cell datasets. It consists of single-cell transcriptome reads from approximately 11 million cells obtained from the middle temporal gyrus and the dorsolateral prefrontal cortex regions of the brain in dementia pathological condition, thus allowing investigation of cellular heterogeneity, gene expression patterns at a single cell level, and their roles in various pathological conditions.

## 3 Results

### 3.1 Organ/tissue transcriptome signature of TRP channels

The human TRP channels family has 27 members that are categorized into six subfamilies based on the presence of unique domains and motifs: TRPA (ankyrin), TRPC (canonical), TRPM (melastatin), TRPML (mucolipins), TRPP (polycystins), and TRPV (vanilloids), ranging from one to eight members as shown on a schematic diagram on the Supplementary Figure S1. Gene ontology analysis recognizes the TRPs’ primary involvement as stimulus-sensing ion (calcium) channels (Supplementary Figure S2A), and the scrutiny of TRP-related biological processes highlights the regulation of biological processes such as response to stimuli or localization (Supplementary Figure S2B). The TRPs as sensors are thus predictably located primarily in membranes and sub-cellular membranous structures, including vesicle-related entities, and their molecular function is defined as calcium ion transporters and molecular binders (Supplementary Figures S2C,D). TRPs are large, multi-domain proteins whose encoding genes are distributed across chromosomes (Supplementary Figure S2E), possessing a complex multi-exonic and multi-isoform structure (Supplementary Figures S2F,G). The TRP genes have larger-than-average transcript/coding sequence length and genomic span (Supplementary Figures S2H,J), while their UTRs’ length and GC content fall within the typical range (Supplementary Figures S2K,M).

**Figure 1:**
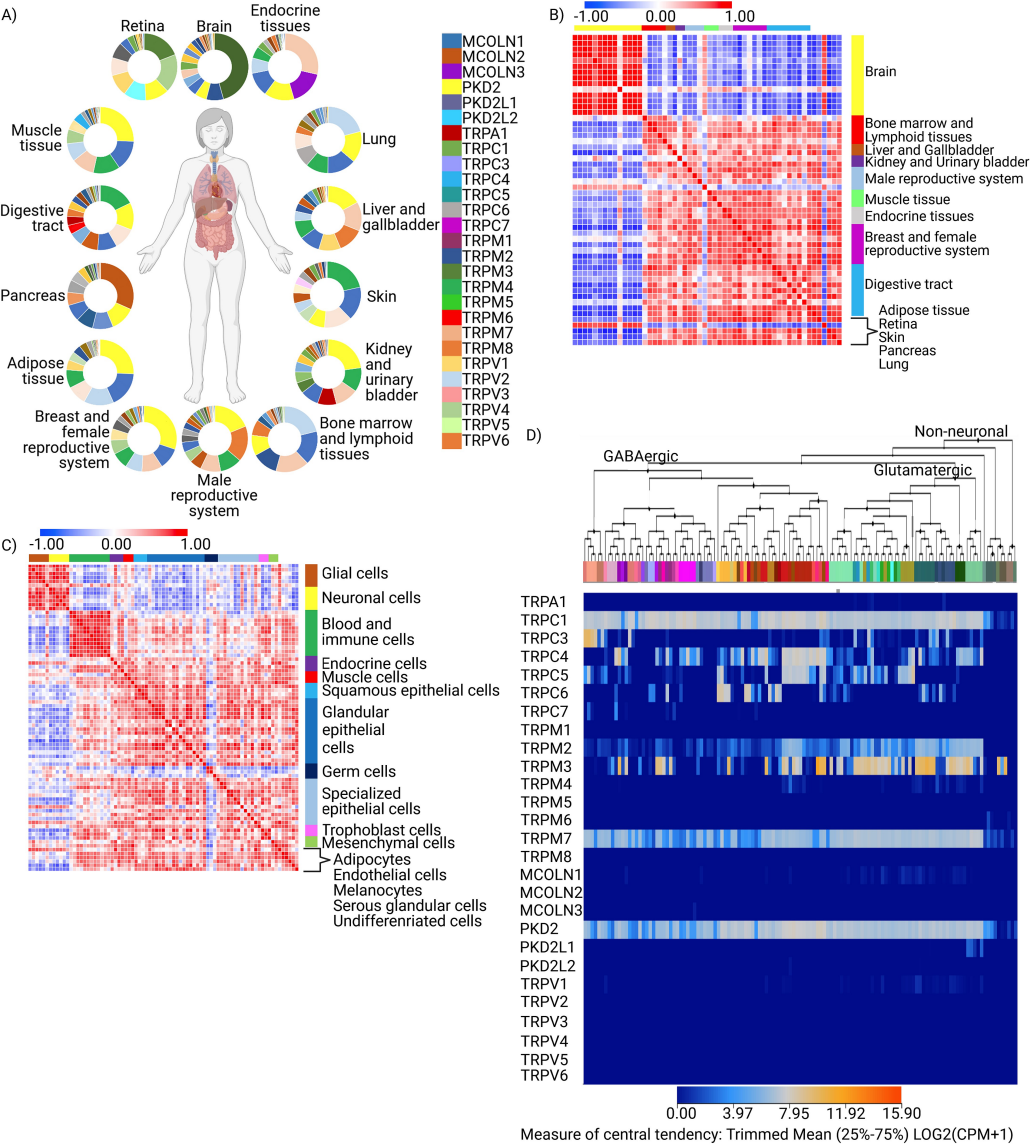
Expression signature of TRP family genes in human organs, tissues, and cells. **(A)** Ring plot showing gene enrichment analysis for the TRP gene family (*n* = 27) in human organs (*n* = 14), generated using the Human Protein Atlas. Method: Normalized read counts (log2). **(B)** Similarity matrix of human organs (*n* = 14) and tissues (*n* = 52), computed as the cross-distance matrix of TRP gene expression (*n* = 27). Method: Pearson correlation. **(C)** Similarity matrix of human cells (*n* = 79) and cell groups (*n* = 16), computed as the cross-distance matrix of TRP gene expression (*n* = 27). Method: Pearson correlation. **(D)** Supervised hierarchical clustering of TRP gene expression (*n* = 27) signatures in neuronal (*n* = 116) and non-neuronal (*n* = 11) cell types. The analysis was performed on a dataset of 4,020 differentially expressed genes from 23,822 cells in the human forebrain using the Allen Brain Map. Method: Median cluster expression, metric—central, linkage method—complete (log2).

Based on the comprehensive query of databases, TRPs exhibit strong organ-specific expression patterns, with only a few members, such as PKD2, being ubiquitously expressed. In contrast, others are differentially expressed across organs (Figure 1A). Particularly distinct expression patterns were observed within the brain (Figure 1B), with TRPM2, PDK2, TRPM7, and TRPC1 strongly expressed and superbly enriched expression of TRPM3 (Figure 1A; Supplementary Figure S3). Interestingly, some of the TRP genes, both strongly (e.g., TRPM2) and weakly (e.g., TRPC3, 5, 7) expressed, are characterized by robust co-occurrence patterns in the brain and within glial/neuronal compartments as measured by similarity matrices (Supplementary Figures S7A,B). Notably, those perceivable brain-specific patterns of expression and co-expression within the TRP family are apparent in both neuronal and glial compartments (Figure 1C; Supplementary Figure S4) and within GABAergic and glutamatergic neuronal sub-populations (Figure 1D), a pattern also perceivable in the mouse brain (Supplementary Figure S5A), highlighting their importance in the overall neuronal function and pointing toward vertebrate-conserved mechanisms. The high expression of most TRP channel types in glutamatergic excitatory neurons and lower expression, with few exceptions (TRPM8, TRPC6, and TRPV6), by inhibitory GABAergic neurons suggests TRP channel involvement in transducing calcium signals in these cell populations (Supplementary Figure S5B). Thus, the distinct TRP gene expression profiles resoundingly differentiate between cells from various organs/tissues, with brain cells displaying the most distinguishable TRP signatures within both glial and neuronal cellular compartments. These features suggest the pivotal role TRPs play in the brain as a significant category of sensory ion channels.

### 3.2 TRP transcriptome signature in brain structures

**Figure 2:**
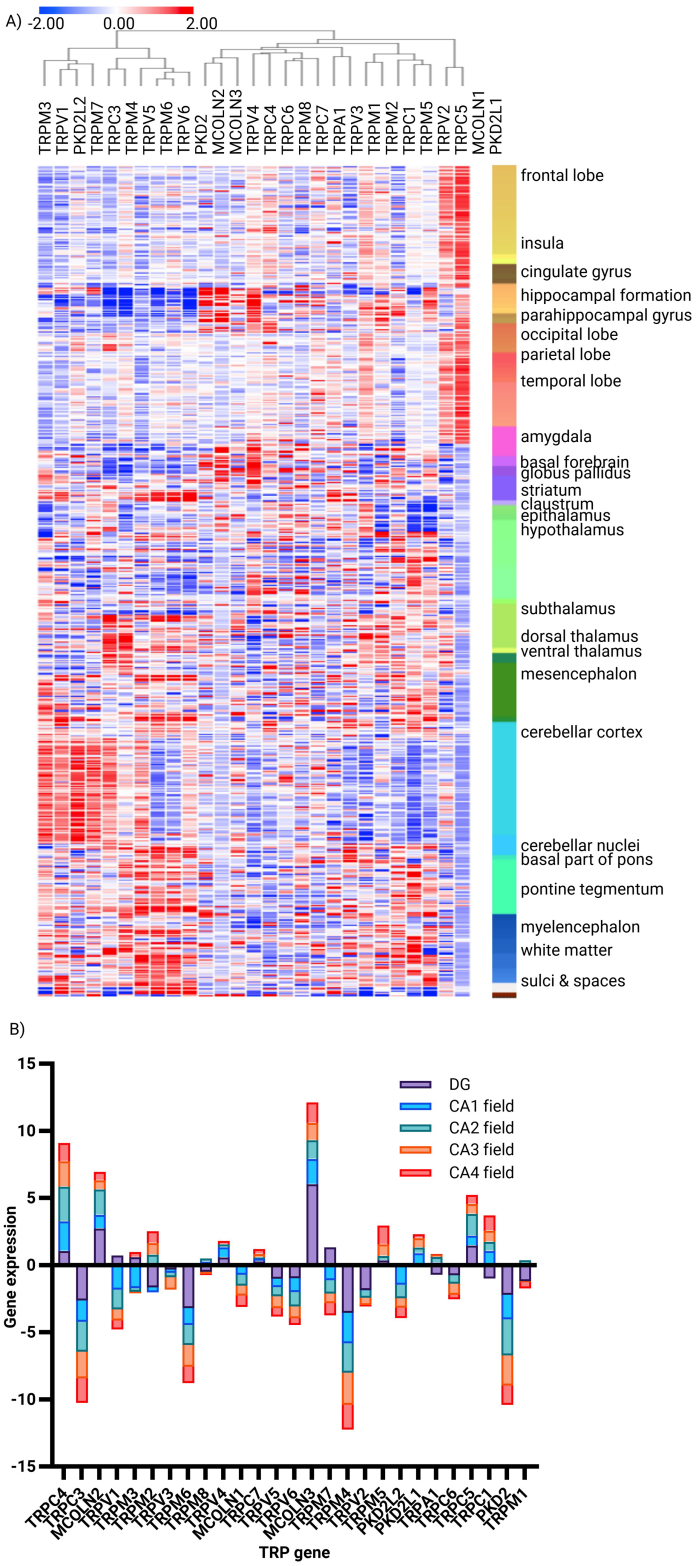
Expression signature of TRP family genes in human brain structures. **(A)** Unsupervised hierarchical clustering of TRP gene expression (*n* = 27) signatures in human brain structures (structures *n* = 160, donor *n* = 6), generated using the Human Protein Atlas. Method: Metric—one minus Pearson, linkage method—complete (*z*-score). **(B)** Bar plot of the most and least enriched TRP genes (*n* = 27) in the human hippocampus, based on gene enrichment analysis using the Human Brain Atlas. Method: Normalized read counts (*z*-score).

The Allen Brain Atlas study focuses on the TRP gene expression profiles in different brain structures, and it is apparent that many exhibit specific enrichment/depletion patterns. Prominent among them is the hippocampus, where the expression of TRPC4 and TRPC5, along with MCOLN2 and MCOLN3, is prevalent, while TRPC3, TRPM6, TRPM4, and PKD2 are depleted (Figure 2A). Several co-expression clusters are apparent across these anatomical regions, exemplified by MCOLN1 and PKD2L1, which are prevalent in all cerebral cortex lobes (Supplementary Figure S7C). Within this particular structure, further gene-specific patterns are also clearly recognizable, with MCOLN3 highly expressed throughout the whole hippocampus. At the same time, TRPC4 is suppressed in the dentate gyrus but prevalent in CA1-4, TRPC5 is evident only in CA2, and MCOLN2 is in CA1-2 and the dentate gyrus. Conversely, TRPM4, PKD2, and TRPC3 are depleted throughout the region, while TRPM6 is particularly strongly suppressed in DG (Figure 2B; Supplementary Figures S6A,B). These observations thus highlight highly regional- and even sub-regional-specific expression patterns of TRP genes, suggesting specialized roles of TRP channels in neural circuitry.

### 3.3 Regulation of TRP transcriptome during brain development

**Figure 3:**
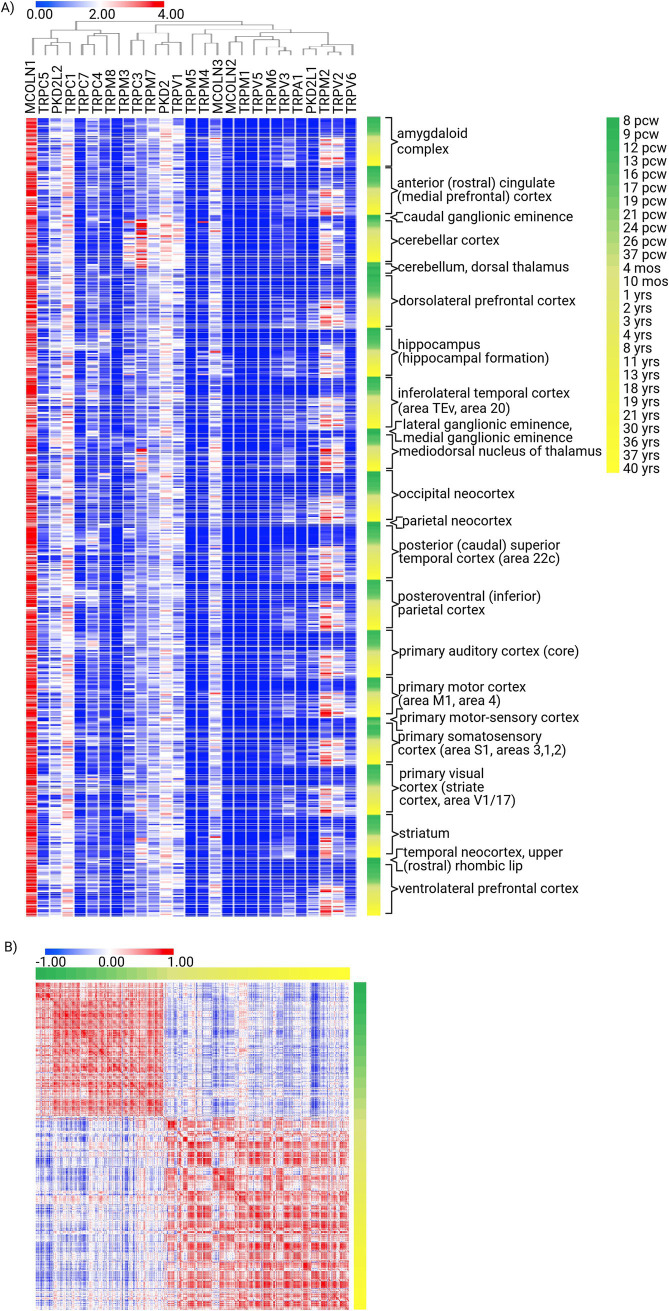
Expression signature of TRP family genes in the structures of the human developing brain. **(A)** Unsupervised hierarchical clustering of TRP gene expression (*n* = 27) signatures in human brain structures during development [8 weeks post-conception (pcw) to 40 years old; structures *n* = 25, donors *n* = 41], generated using the Developing Human Brain Atlas. Method: Metric—one minus Pearson correlation, linkage method—complete (log2). **(B)** Similarity matrix of TRP gene expression (*n* = 27) in human brain structures during development (*n* = 25), computed as the cross-distance matrix. Method: Pearson correlation.

The Allen Brain Developmental Atlas study demonstrates the tightly regulated expression of TRP genes during brain development. To underline the most apparent patterns, it is evident that while TRPM4, TRPC1, and, above all, MCOLN1 show ubiquitous and highly expressed profiles regardless of the developmental stage, TRPV2, TRPM2, and TRPV3 expression patterns are developmental stage-dependent (i.e., absent in fetal samples while prevalent in postnatal ones). Thus, multiple spatiotemporal patterns of expression can be identified for TRP genes in the brain: ubiquitously and continuously expressed, almost non-existent, region-specific regardless of time (e.g., TRPC3 in cerebellar cortex), and region-specific in a developmental stage-specific manner (Figure 3A; Supplementary Figure S7D). Holistically, though, the TRP genes are characterized by developmental stage-dependent expression, as evident from the similarity matrix, with a robust perinatal turning point (Figure 3B). Thus, it can be hypothesized that those developmental-specific TRPs become crucial upon birth by acquiring the independent sensory experiences of external stimuli.

### 3.4 TRP transcriptome signature in aging brain and dementia

**Figure 4:**
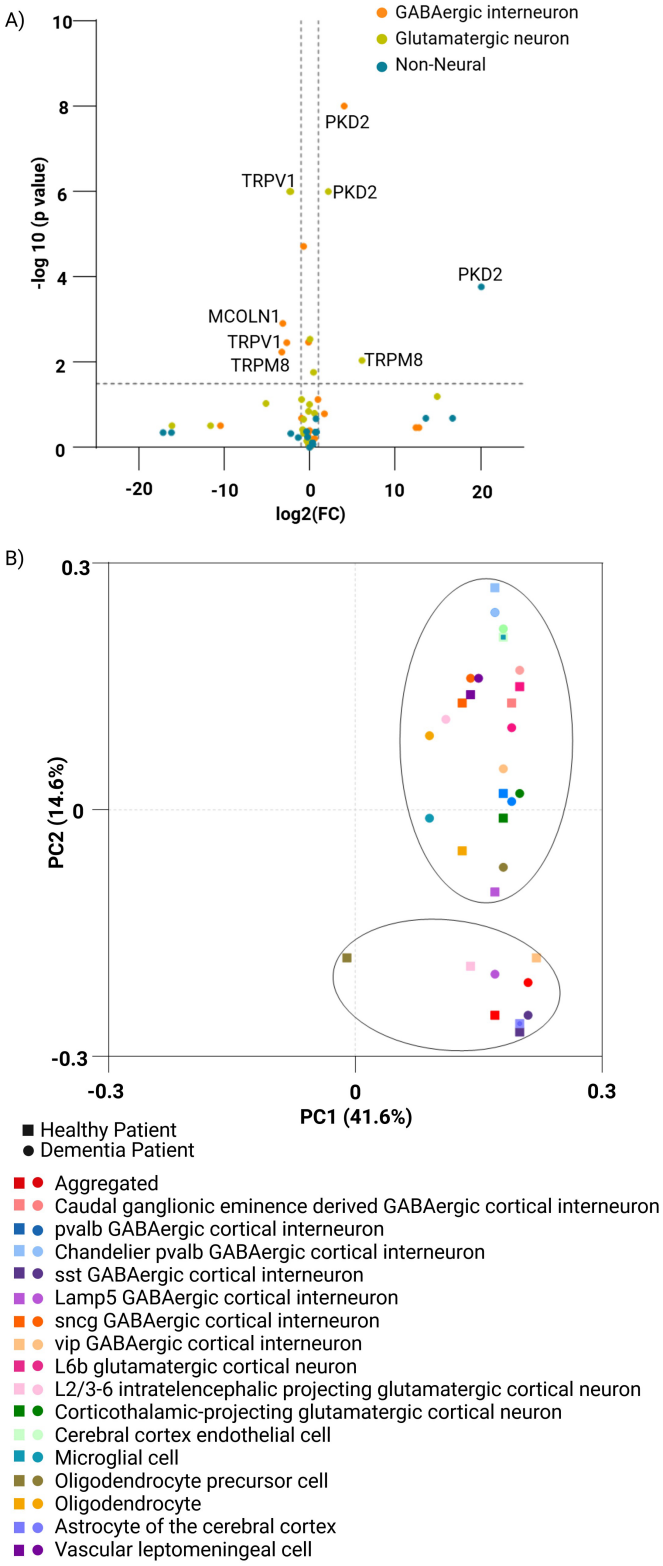
Comparison of the expression of TRP family genes across human normal and dementia brain cells. **(A)** Volcano plot of TRP gene expression (*n* = 27) comparing neuronal and non-neuronal cell types in the human forebrain under healthy and dementia conditions. Healthy samples (*n* = 23,822 single-nucleus transcriptomes) were analyzed based on 4,020 differentially expressed genes, derived from the Allen Brain Map. Dementia samples originate from post-mortem brain specimens (*n* = 5) and include 166,868 single-nucleus transcriptomes from The Seattle Alzheimer’s Disease Brain Cell Atlas (SEA-AD). **(B)** PCA plot using the ClustVis platform generated by the study for the family of TRP genes (*n* = 27) in a set of cells from healthy (*n* = 11,164,441) and dementia (*n* = 1,052,021) patient brains covering different cell types from the middle temporal gyrus and the dorsolateral prefrontal cortex regions of the brain (*n* = 17) feature using CZ CELLxGENE Discover Platform. Principal components PC1 and PC2 explain 41.6 and 14.6% of the variance, respectively. Distinct symbols and colors represent brain cell types, while healthy and dementia samples are indicated by square and circular markers, respectively.

The Seattle Alzheimer’s Disease Brain Cell Atlas study compared the TRP gene expression profiles in post-mortem brain specimens from the forebrain of healthy individuals and dementia patients on a single-nucleus transcriptome level. It was apparent that while most TRP genes were not altered by dementia, few (most significantly PKD2) were consistently down-regulated in all studied cell subpopulations of dementia brains, a handful was upregulated there (TRPV1, in both glutamatergic and GABAergic neurons, but not in non-neuronal cells), all the while some were altered in cell type-specific patterns (e.g., TRPM8 downregulated in dementia glutamatergic neurons while upregulated in GABAergic ones) (Figure 4A; Supplementary Figure S8A).

Single-cell analysis reveals cell-type-specific expression patterns of TRP genes, with notable shifts in TRP gene expression observed in some sub-populations of dementia patients’ brains (harvested from the middle temporal gyrus and the dorsolateral prefrontal cortex), indicative of pathology-driven changes. The principal component analysis showed that while most sub-populations were not affected in the demented brain, others were significantly separated, most notably in oligodendrocytes’ precursors as well as in some GABAergic cortical interneurons (Lamp5 and vip), thus highlighting the importance of considering cellular heterogeneity when studying TRP channel involvement in dementia, and suggesting a potential impact on inhibitory neurotransmission in the process (Figure 4B; Supplementary Figures S8B,E).

Finally, the brain’s TRP expression analysis revealed regional patterns, most pronounced in the hippocampus and the white matter of the forebrain (Supplementary Figure S8C); however, granular analysis failed to detect significant dementia-related alterations at the brain region level (Supplementary Figures S8D, S9A,B). In summary, these findings provide insights into the spatial and temporal regulation of TRP gene expression in the brain, shedding light on their biological significance and potential implications for therapeutic interventions in sensory disorders and cognitive decline.

The results of our analysis suggest that TRP channel expression is altered in both aging and dementia, with specific changes observed in different brain regions and cell types. These alterations may contribute to the cognitive decline observed in these conditions. Further research is needed to determine the functional consequences of these expression changes and to explore the potential of targeting TRP channels for therapeutic interventions in age-related cognitive decline and dementia.

## 4 Discussion

The discovery of transient receptor potential (TRP) channels over five decades ago marked the beginning of a profound expansion in our understanding of cellular signaling mechanisms that govern a plethora of physiological processes. From the initial observations in model organisms to the detailed characterization of the TRP superfamily in humans, our comprehension of these channels has evolved to appreciate their ubiquity and functional diversity across a wide range of biological systems.

The historical context provided by the seminal works of Cosens and Manning (1969) and later by Montell and Rubin (1989) underscored the importance of TRP channels in sensory perception. Subsequent research expanded the family to include 27 mammalian genes categorized into six major subfamilies. The recognition of their role in physiological processes ranging from temperature sensation to the regulation of calcium homeostasis grew over time, culminating in the awarding of the Nobel Prize in Physiology or Medicine in 2021, highlighting the significant impact of these studies on biomedical sciences.

Our current results elaborate on this foundation by detailing the organ- and tissue-specific expression patterns of TRP channels, reinforcing the concept that these channels are not only pervasive but also tailored to meet the demands of specific cellular contexts. The expression patterns observed in various human organs, particularly in brain regions, align with the functional requirements of these tissues.

The strong presence of TRP channels in the brain indicates their involvement in the complex mechanism of intracellular Ca^2+^ signaling, which is crucial for the organ’s homeostasis and functionality. TRP channels mediate slow excitatory signaling resulting from mGluR activation and modulate GABA and glutamate transmission (Topolnik et al., 2006; Ranjbar et al., 2022). The high expression of TRP channels in excitatory glutamatergic neurons was thus anticipated, as the excitotoxic transmission is based mainly on calcium currents. Conversely, the apparent lower expression of these channels in inhibitory GABAergic neurons can be explained by the reliance of GABA receptors on Ca^2+^ currents independent of G protein signaling (Kubota et al., 2014).

Ionotropic glutamate receptors supported by voltage-operated calcium channels play a central role in Ca^2+^ fluxes, triggering pathways responsible for brain development and plasticity, dysfunction, and neurodegenerative diseases. Evidence suggests TRP channel involvement (Egbenya and Adjorlolo, 2021; Sawamura et al., 2017; Charles et al., 2024). For example, the enrichment of TRPC4 and TRPC5 in the hippocampus and their roles in dendritic and synaptic functions exemplify the specialized adaptations of TRP channels to support cognitive functions and neural plasticity.

The differential expression patterns across development stages and somewhat distorted expression among different pathological conditions, such as dementia, provide insights into the dynamic regulation of TRP channels in response to developmental cues and disease initiation and progression. The developmental regulation of TRP channel expression TRPV2, TRPM2, and TRPV3, or TRPC3-specific expression in the cerebellar cortex, and their modulation in aging and dementia, underscore their potential roles in developmental neurobiology and neurodegenerative diseases, respectively, as it has already been shown that the dysfunction of TRP channels localized in the brain contributed to Alzheimer’s disease (AD), Huntington’s disease (HD), Parkinson’s disease (PD), and Amyotrophic lateral sclerosis (LS) (Rather et al., 2023). Their role in learning and memory formation may also explain memory impairment accompanying these diseases (Krolik et al., 2020; Gualdani and Gailly, 2020). Thus, changes in expression patterns observed in the aging/dementia brain may reflect both contributory and compensatory mechanisms in disease progression.

In summary, the expanded understanding of TRP channels provided by both historical perspectives and current research underscores their significance in a broad array of biological functions. These findings support ongoing efforts to target TRP channels in therapeutic interventions to modulate their activity in various diseases. The continuous exploration of TRP channels promises to enhance our understanding of cell physiology and open new avenues for treating diseases where these channels play a pivotal role.

## 5 Summary

The presence and distribution of TRP channels in the human body generate many questions concerning their involvement in cellular homeostasis. The significance of the differential expression of TRP genes in different biological contexts is still poorly comprehended, as mutations or abnormal expression of TRP channels are associated with a broad spectrum of diseases, ranging from neurodegenerative diseases, diabetic nephropathy, breast cancer, to Duchenne muscular dystrophy. This paper applied differential co-expression analysis of structural and functional properties to grasp the TRP family gene regulation better and provide a foundation for envisioning new therapeutic strategies. We argue that, being large and complex proteins, the TRP family members are primarily controlled at the transcriptional level, thus rendering the transcriptomic approach informative and sufficient to comprehend their patterns of (co)expression and, therefore, activity.

The TRP family genes are pivotal for sensory perception and the maintenance of cellular homeostasis. Dysregulated TRP gene expression can lead to sensory impairments and cognitive dysfunction, notably in neurodegenerative disorders like dementia. This study investigates the relationship between TRP gene expression and dementia, identifying altered expression in various cell subpopulations of the demented brain, potentially implicating these changes in sensory impairments. Differential clustering patterns in some of these subpopulations in dementia suggest the significance of TRP gene dysregulation in sensory disability pathophysiology. These findings thus open avenues for further research and therapeutic interventions targeting TRP gene expression to ameliorate impairments in dementia patients and highlight the regulatory dynamics and biological significance of TRP gene expression in the brain.

## Data Availability

The original contributions presented in the study are included in the article/Supplementary material, further inquiries can be directed to the corresponding author.

## Footnotes

^1^ https://software.broadinstitute.org/morpheus

^2^ https://www.proteinatlas.org/

^3^ https://www.proteinatlas.org/humanproteome/tissue

^4^ https://www.proteinatlas.org/humanproteome/tissue+cell+type

^5^ https://portal.brain-map.org/

^6^ https://human.brain-map.org/

^7^ https://portal.brain-map.org/atlases-and-data/bkp/abc-atlas

^8^ https://www.brainspan.org/

^9^ https://celltypes.brain-map.org/

^10^ https://aging.brain-map.org/

^11^ https://portal.brain-map.org/explore/seattle-alzheimers-disease

^12^ https://cellxgene.cziscience.com/
