# Supplementary material for: Transcriptomic analysis of the TRP gene family in human brain physiopathology
Source: Front Mol Neurosci. 2025 Apr 24;18:1576941. doi: 10.3389/fnmol.2025.1576941 (PMC12058757; doi:10.3389/fnmol.2025.1576941)
Supplement: Supplementary file 2 [file Data_Sheet_2.docx]

Supplementary Material

## Supplementary Figures


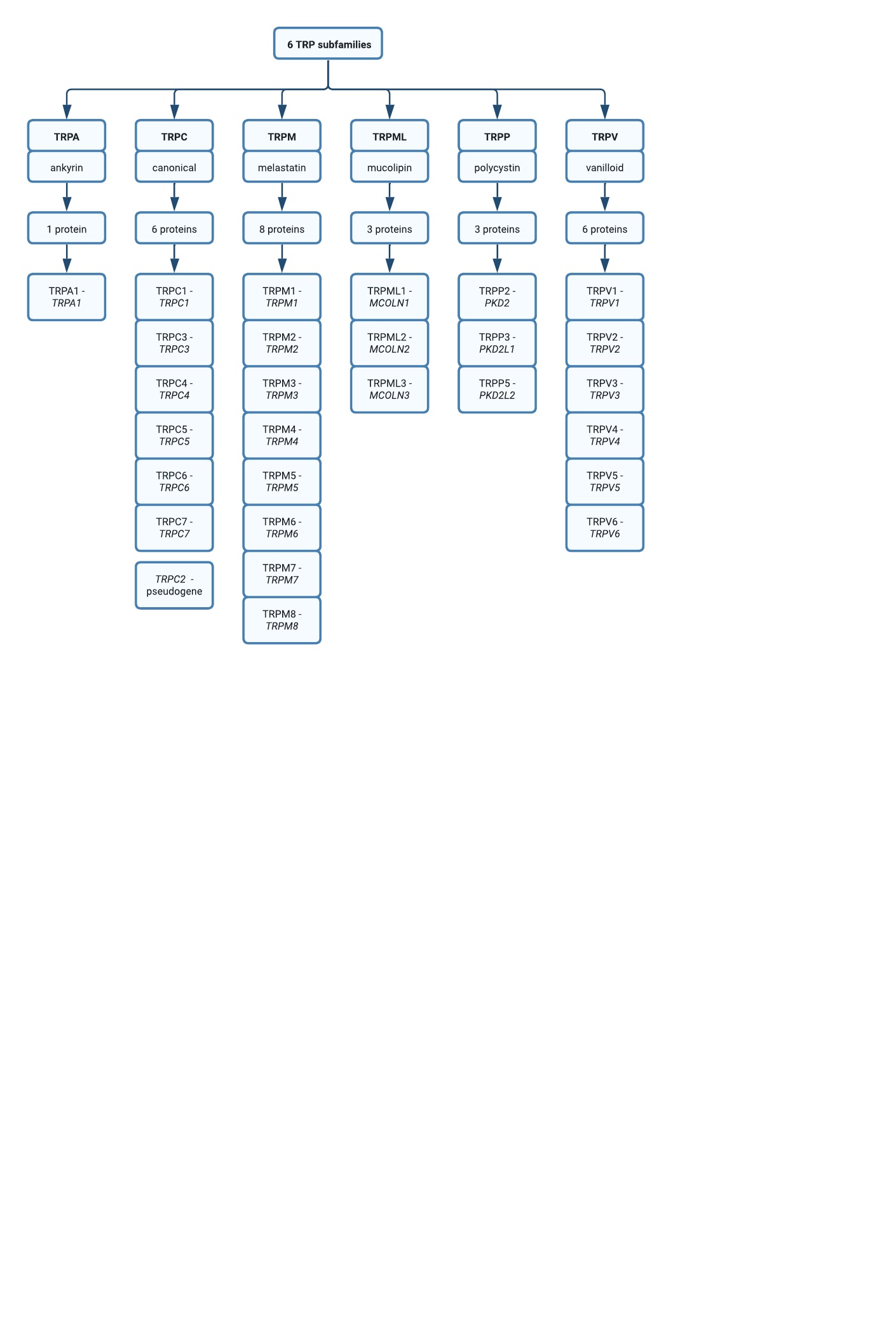


**Figure S1. Human TRP gene family members’ tree based on amino acid sequence homology.**


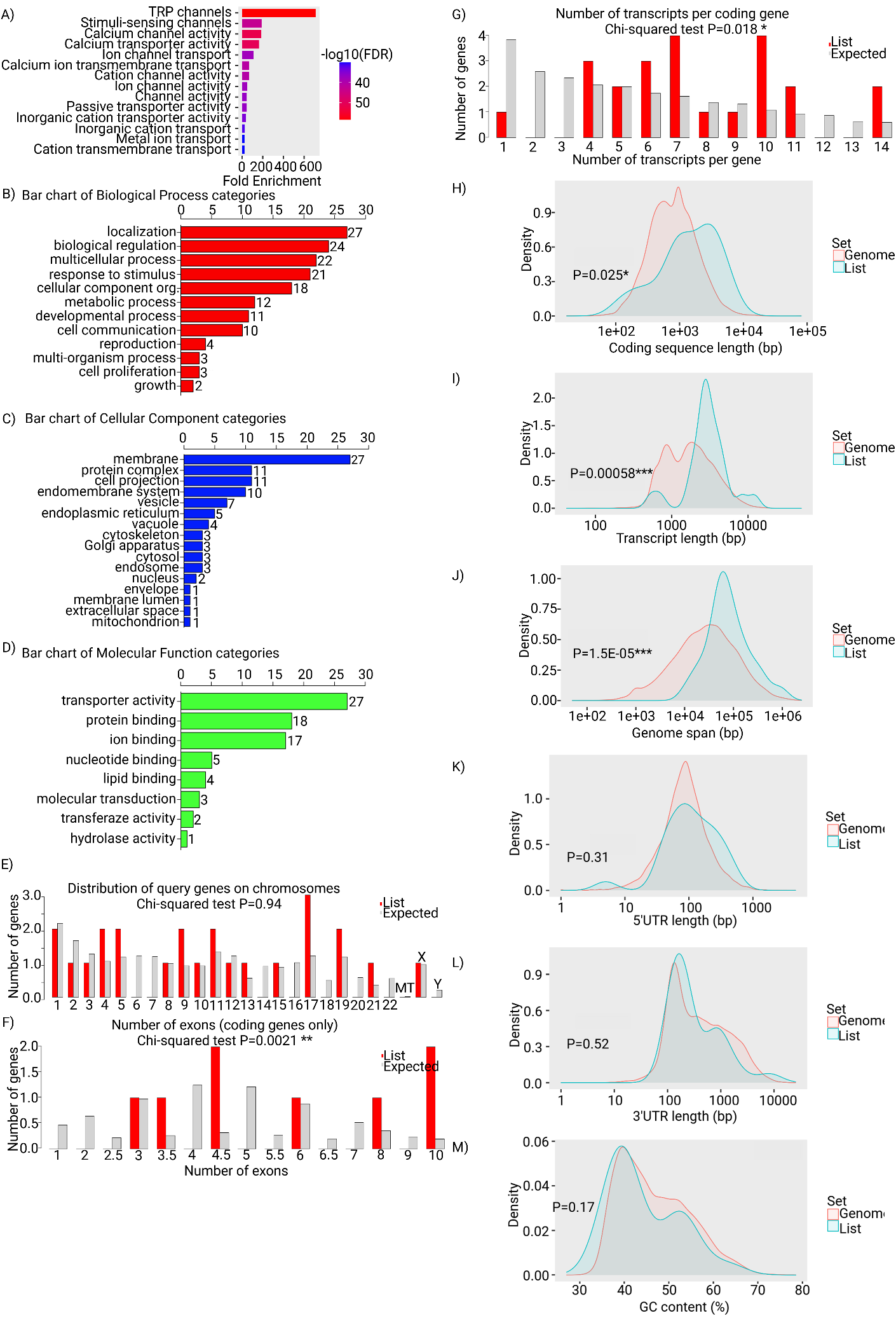


**Figure S2. Functional annotation analysis and comparative characteristics of TRP family genes against the human transcriptome.**
a) Bar plot of gene ontology (GO) enrichment analysis for the TRP gene family (n = 27), generated using ShinyGO. **Method:** Enrichment fold, FDR [-log10].
b) Bar plot of biological process (BP) enrichment analysis for TRP genes(n = 27), generated using WebGestalt. **Method:** Enrichment score, FDR [-log10].
c) Bar plot of cellular component (CC) enrichment analysis for TRP genes(n = 27), generated using WebGestalt. **Method:** Enrichment score, FDR [-log10].
d) Bar plot of molecular function (MF) enrichment analysis for TRP genes(n = 27), generated using WebGestalt. **Method:** Enrichment score, FDR [-log10].
e) Bar plot of TRP gene(n = 27) distribution across chromosomes, generated using ShinyGO. **Method:** Chi-squared test.
f) Bar plot of TRP gene(n = 27) distribution by exon number, generated using ShinyGO. **Method:** Chi-squared test.
g) Bar plot of TRP gene(n = 27) distribution by transcript number, generated using ShinyGO. **Method:** Chi-squared test.
h) Density plot of coding sequence length for TRP genes(n = 27), generated using ShinyGO.
i) Density plot of transcript length for TRP genes(n = 27), generated using ShinyGO.

j) Density plot of genome span for TRP genes(n = 27), generated using ShinyGO.

k) Density plot of 5’ UTR for TRP genes (n = 27), generated using ShinyGO.

l) Density plot of 3’ UTR for TRP genes (n = 27), generated using ShinyGO.

m) Density plot of GC content for TRP genes (n = 27), generated using ShinyGO.


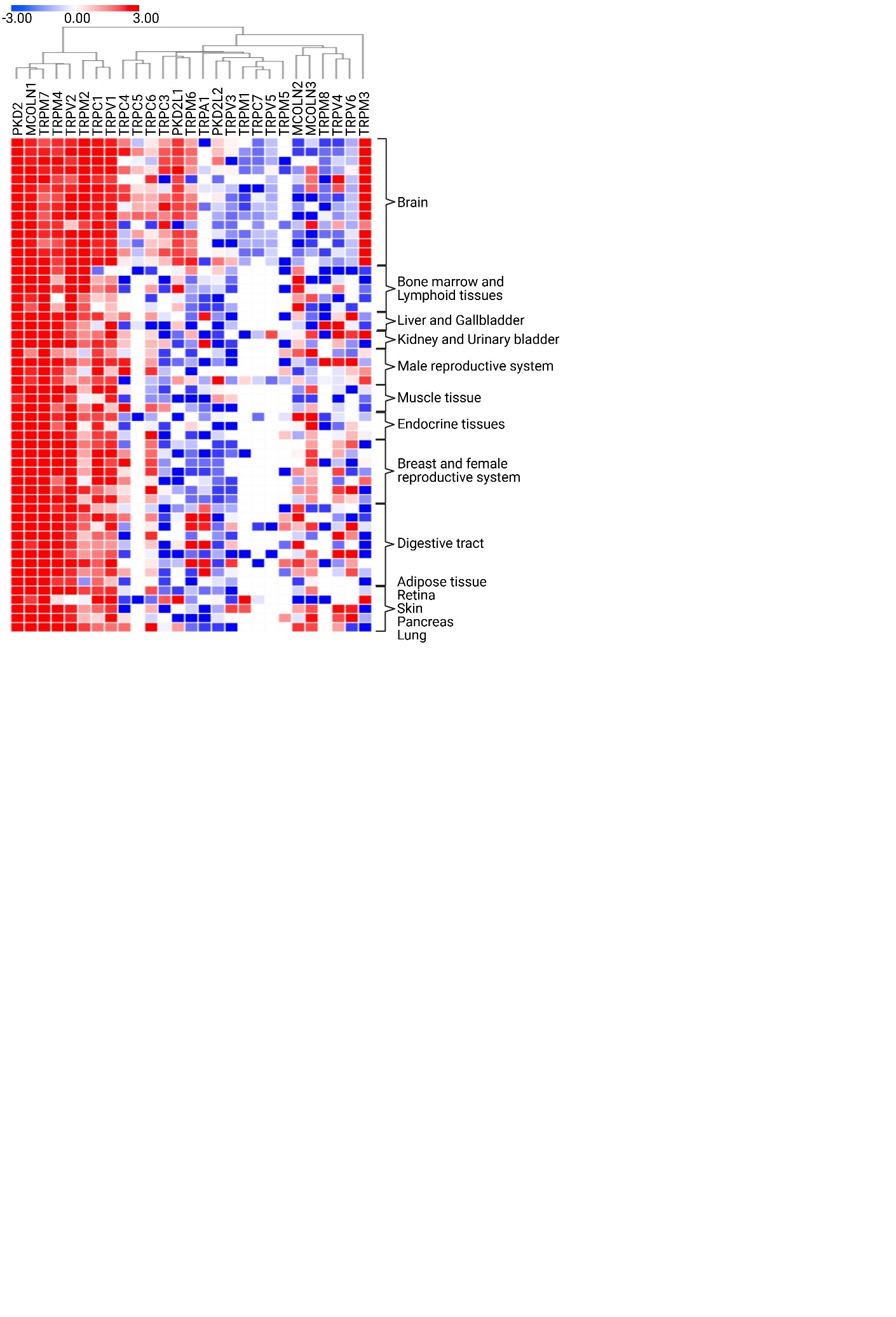


**Figure S3. Transcriptional enrichment of TRP family genes in human organs.**
Unsupervised hierarchical clustering of TRP gene expression (n = 27) in organs (n = 14) and tissues (n = 52), generated using the Human Protein Atlas. **Method:** Metric – Euclidean distance, linkage method – complete [log2].


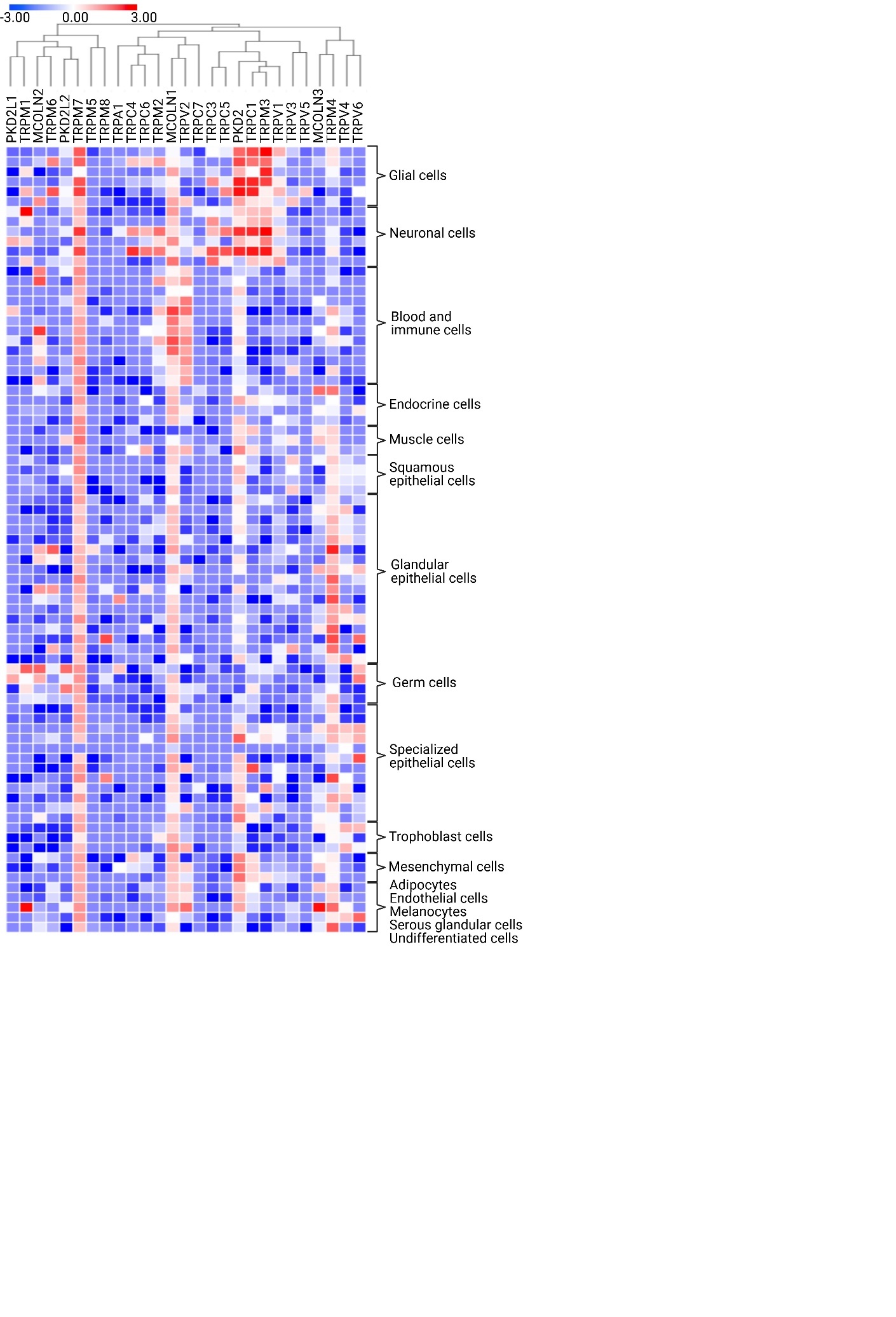


**Figure S4. Expression signature of TRP family genes in human cells.**
Unsupervised hierarchical clustering heatmap of TRP gene expression (n = 27) in cell types (n = 79) and cell subgroups (n = 15), generated using the Human Protein Atlas. **Method:** Metric – one minus Pearson correlation, linkage method – complete [log2].


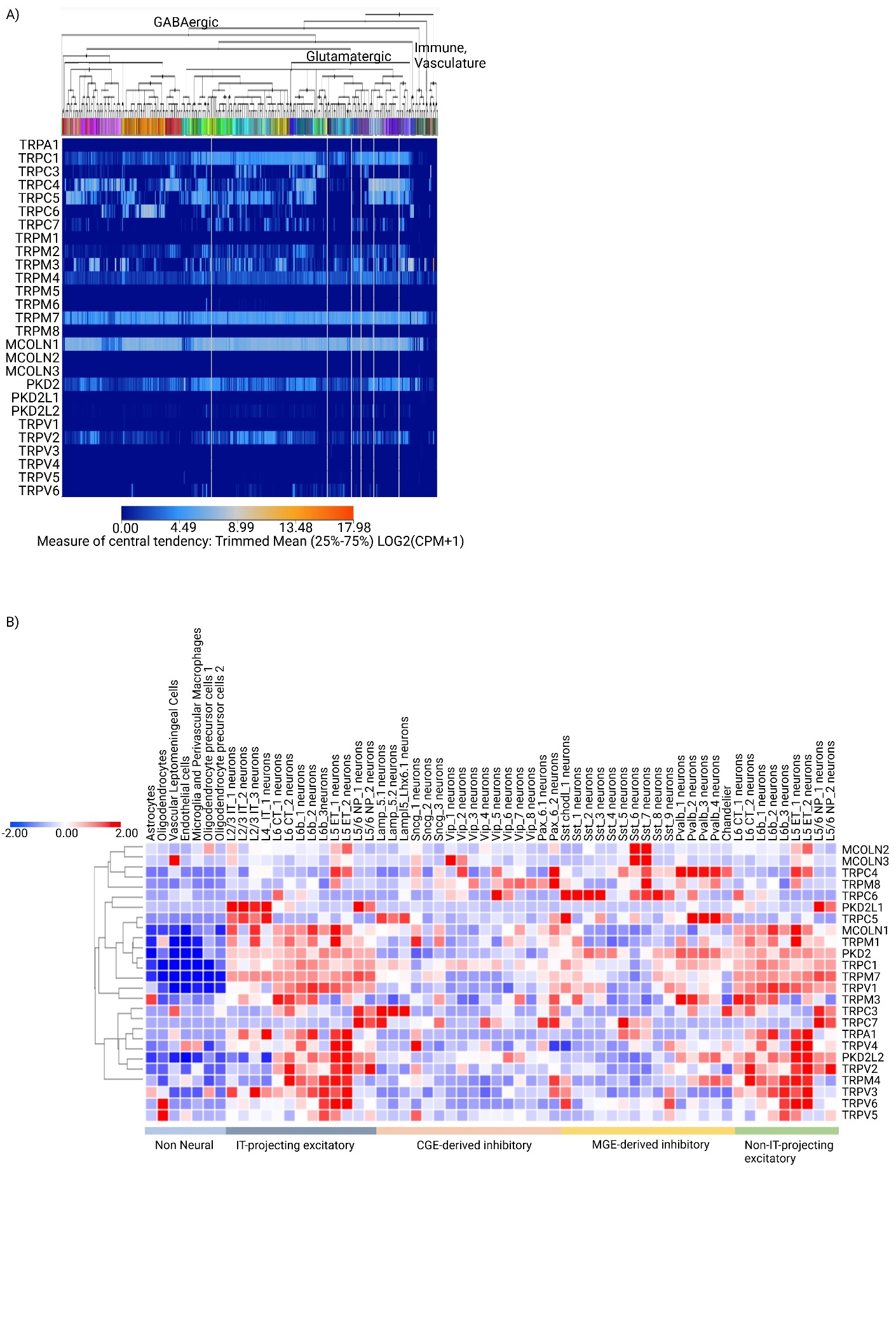


**Figure S5. Association of TRP family genes with transcriptional markers in single-cell analysis of the forebrain.**
a) Supervised hierarchical clustering of TRP gene expression (n = 27) in single-cell transcriptomes from multiple cortical areas and the hippocampal formation (1,093,785 total cells), generated using the Allen Brain Map.
b) Unsupervised hierarchical clustering heatmap of TRP gene expression (n = 27) in single-cell types (n = 60) and subgroups (n = 5: Non-neuronal cells [Non Neural], Intratelencephalic-projecting excitatory (glutamatergic) neurons projecting within the telencephalon [IT-projecting excitatory], Caudal Ganglionic Eminence-derived inhibitory (GABAergic) interneurons [CGE-derived inhibitory], Medial Ganglionic Eminence-derived inhibitory (GABAergic) interneurons [MGE-derived inhibitory], Excitatory (glutamatergic) neurons projecting to subcortical targets [Non-IT-projecting excitatory]) from the middle temporal gyrus (MTG), generated using Cytosplore Simian Viewer. **Method:** Metric – one minus Pearson correlation, linkage method – complete [log2].


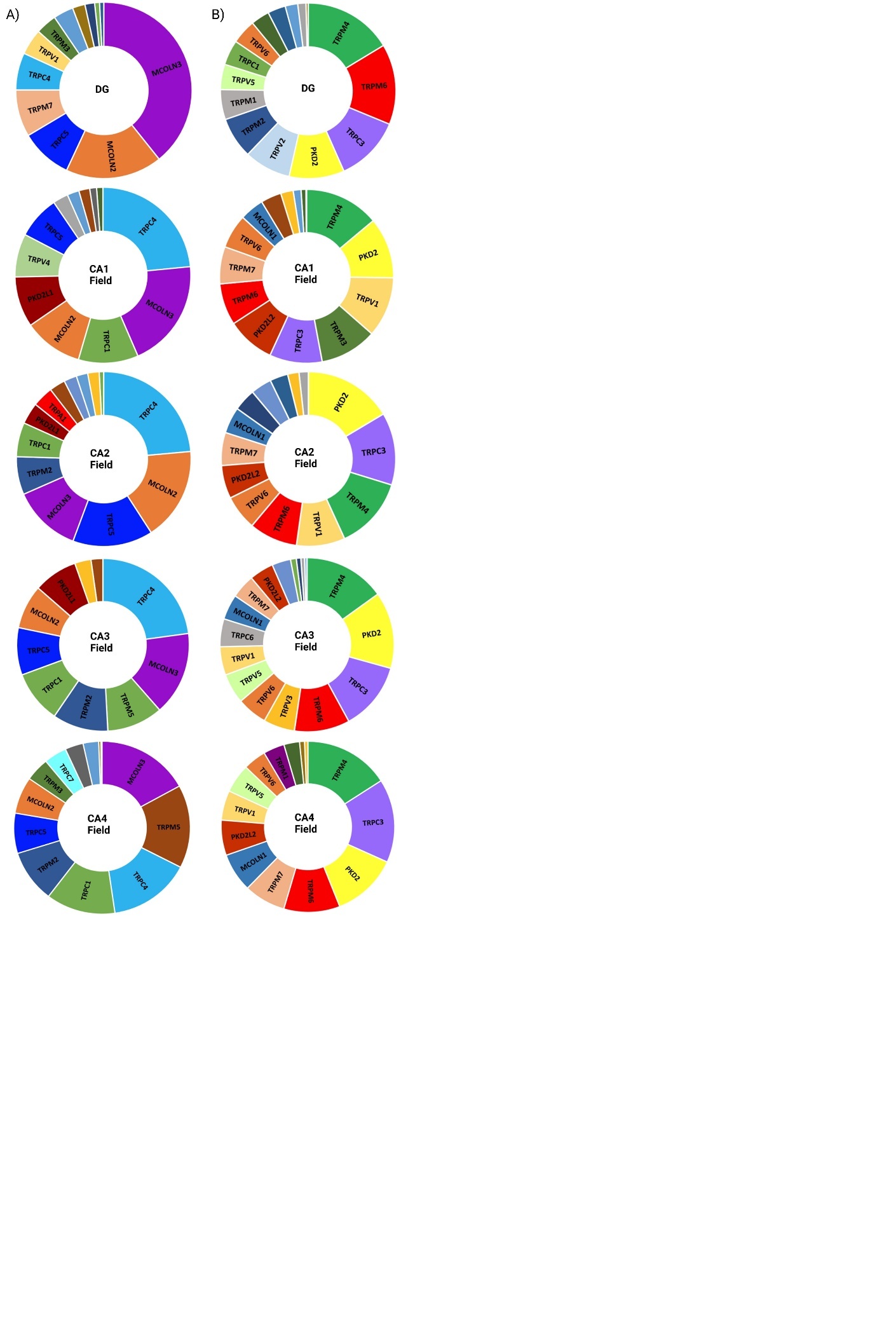


**Figure S6. Transcriptional enrichment of TRP family genes in the human hippocampus.**
a) Ring plot of the most enriched TRP genes (n = 27) in the human hippocampus, generated using the Human Brain Atlas. **Method:** Normalized read counts [log2].
b) Ring plot of the least enriched TRP genes (n = 27) in the human hippocampus, generated using the Human Brain Atlas. **Method:** Normalized read counts [log2].


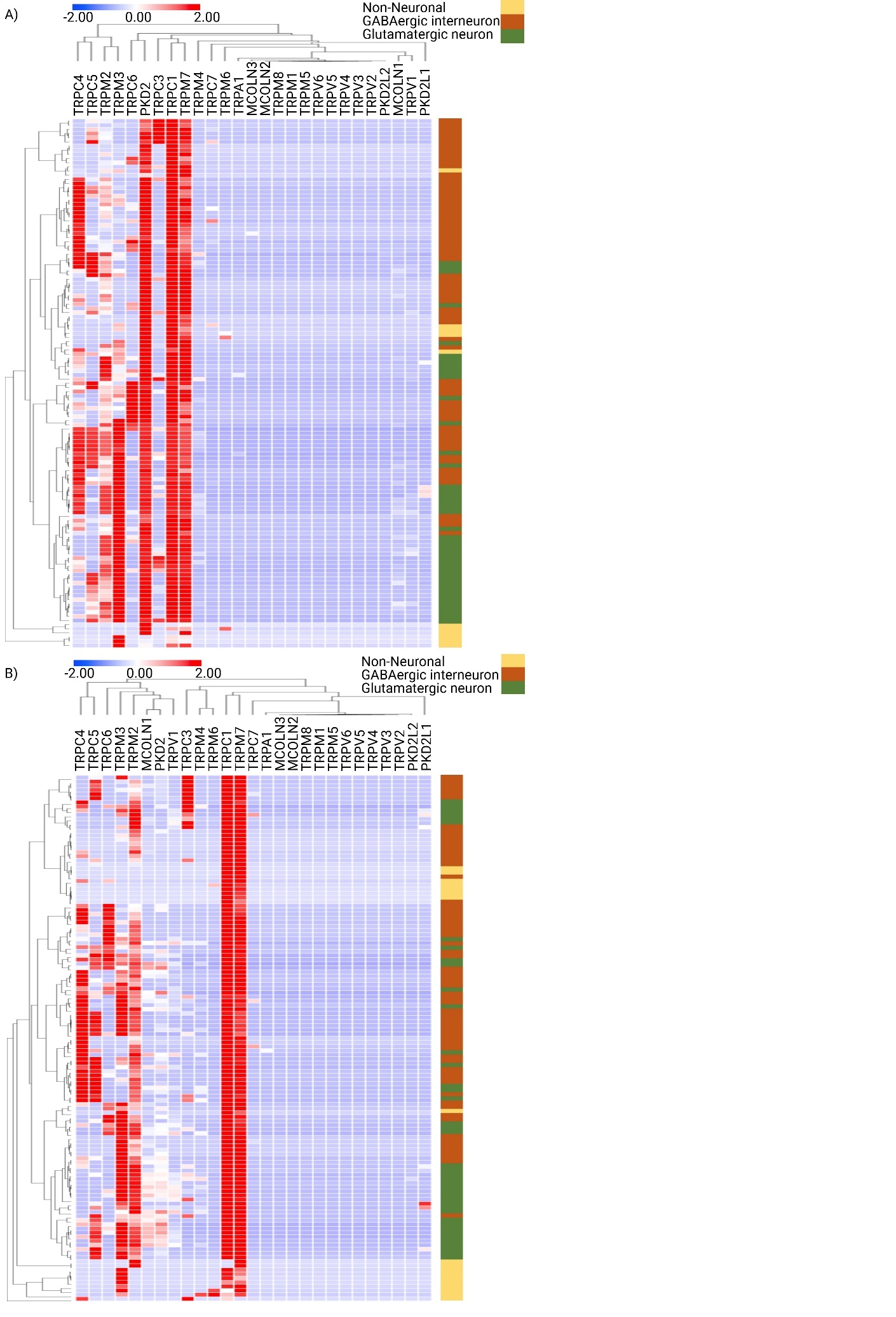


**Figure S7. TRP family gene expression in neuronal and non-neuronal cell types in human hippocampus.**
a) Unsupervised hierarchical clustering of TRP gene expression (n = 27) in neuronal (n = 116) and non-neuronal (n = 11) cell types, based on median cluster expression for 4,020 differentially expressed genes (n = 23,822 cells) in normal brain, generated using the Allen Brain Map. **Method:** Metric – one minus Pearson, linkage method – complete [z-score].
b) Unsupervised hierarchical clustering of TRP gene expression (n = 27) in neuronal (n = 109) and non-neuronal (n = 18) cell types, based on single-nucleus transcriptomes (166,868 total nuclei) from 5 post-mortem dementia brain specimens, generated using The Seattle Alzheimer’s Disease Brain Cell Atlas (SEA-AD). **Method:** Metric – one minus Pearson, linkage method – complete [z-score].


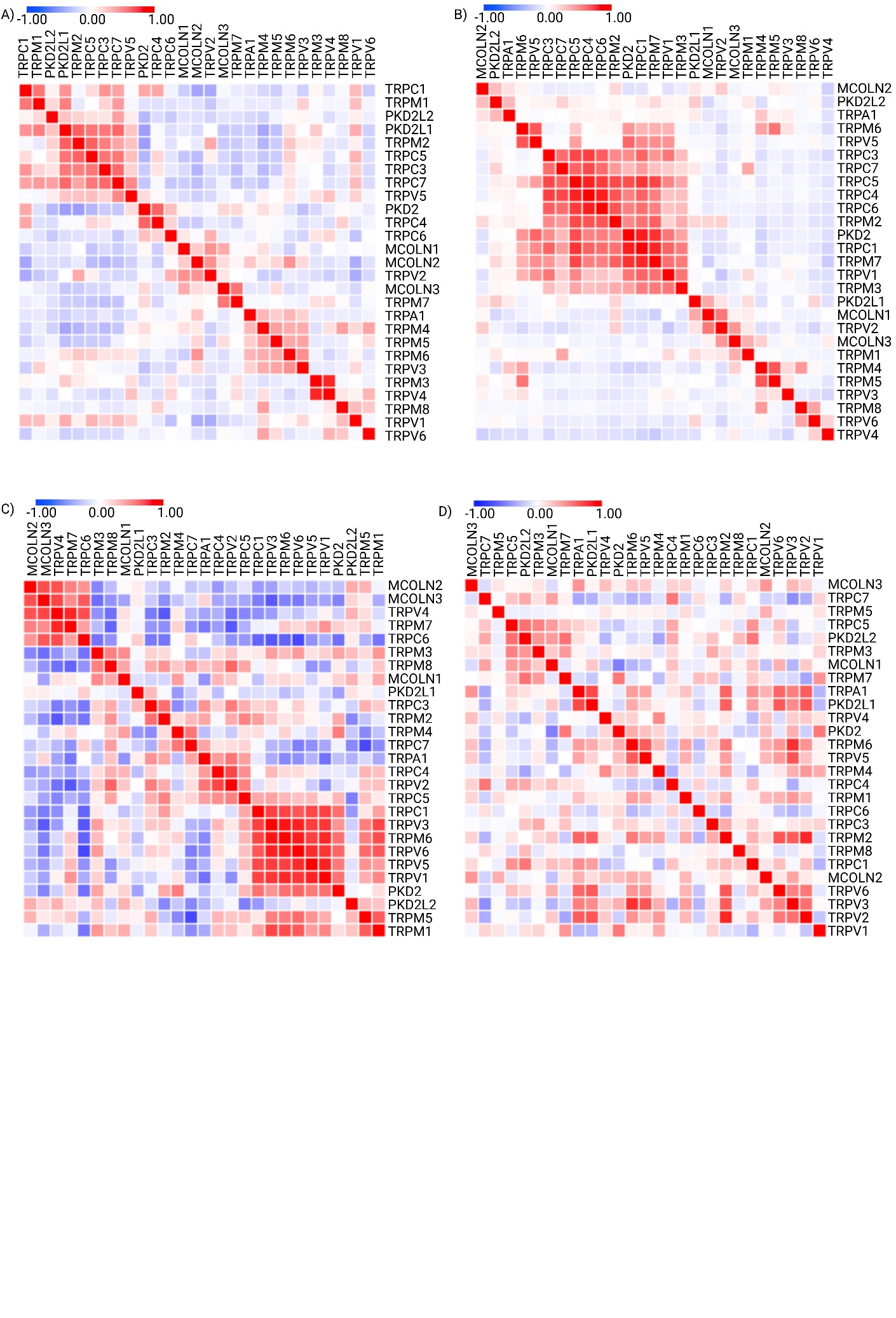


**Figure S8. Similarity matrices of TRP gene expression across human organs, cells, and brain structures at different ages.**
a) Similarity matrix of TRP gene expression (n = 27) in human organs (n = 14) and tissues (n = 52). **Method:** Pearson correlation.
b) Similarity matrix of TRP gene expression (n = 27) in human cells (n = 79) and cell groups (n = 15). **Method:** Pearson correlation.
c) Similarity matrix of TRP gene expression (n = 27) in human brain structures (n = 27). **Method:** Pearson correlation.
d) Similarity matrix of TRP gene expression (n = 27) in human brain structures at different donor ages (n = 41). **Method:** Pearson correlation.


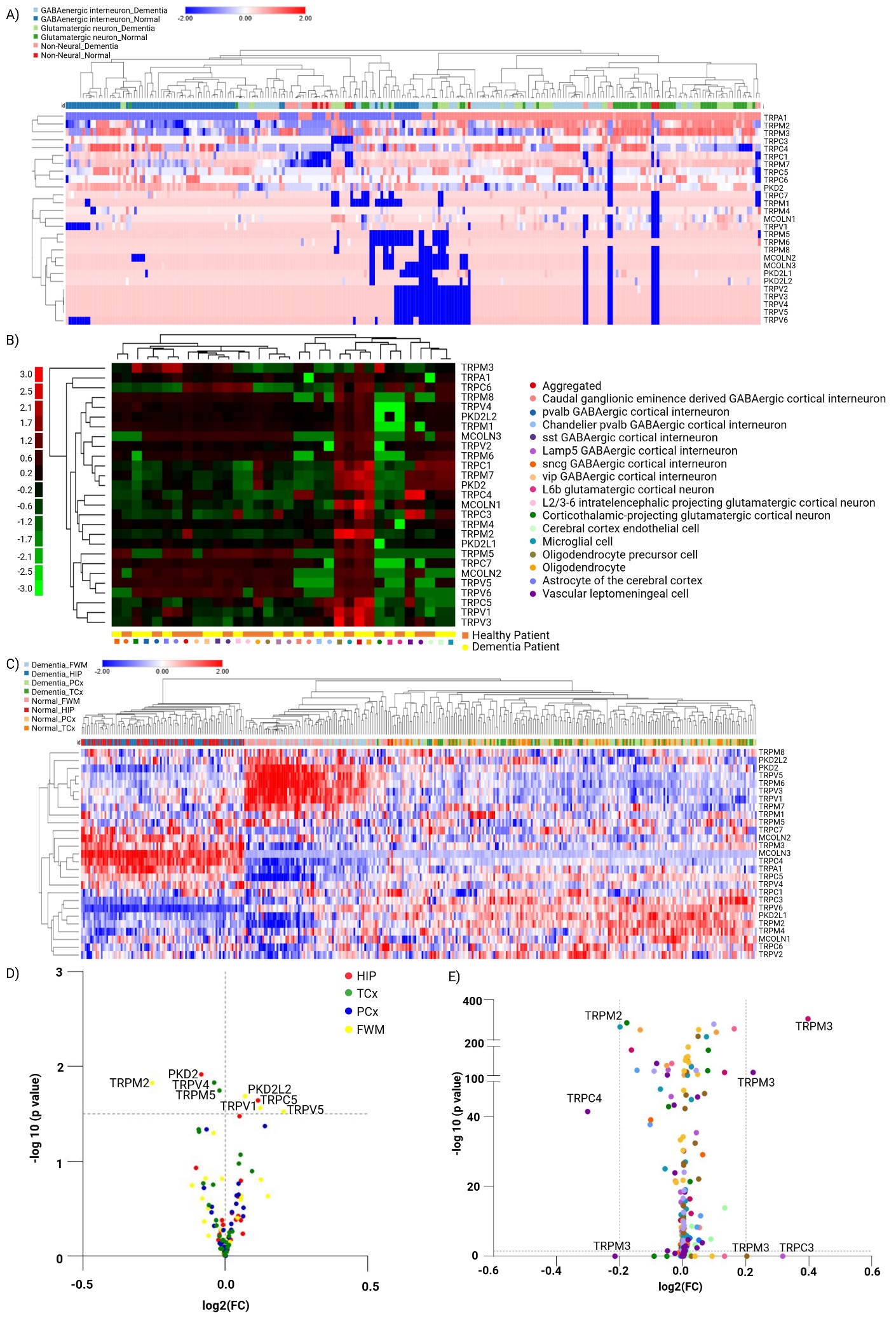


**Figure S9. Comparison of TRP family gene expression across human normal and dementia brain structures and cells**

a) Heatmap of TRP gene expression (n = 27) signatures in neuronal (n = 116, including GABAergic interneurons and glutamatergic neurons) and non-neuronal (n = 11) cell types in the healthy human forebrain, based on 4,020 differentially expressed genes (n = 23,822 cells), generated using the Allen Brain Map. TRP gene expression (n = 27) signatures was analyzed for neuronal (n = 109) and non-neuronal (n = 18) cell types in post-mortem brain specimens from dementia patients (n = 5; single-nucleus transcriptomes, total nuclei n = 166,868), using The Seattle Alzheimer’s Disease Brain Cell Atlas (SEA-AD). **Method:** Metric – Rank correlation, linkage method – average by cluster tightness.

b) TRP gene signatures in normal versus dementia brains. Unsupervised heatmap of TRP gene expression (n = 27) in brain cell types (n = 17) from healthy (n = 11,164,441 cells) and dementia (n = 1,052,021 cells) patients, generated using the CZ CELLxGENE Discover Platform. **Method:** Metric – Rank correlation, linkage method – average by cluster tightness.

c) Heatmap of differentially expressed genes (DEA) for the TRP gene family (n = 27) in human brain structures from an aging cohort study (structures n = 4: hippocampus [HIP], temporal neocortex [TCX], white matter of the forebrain [FWM], piriform cortex [PCX]; donors n = 98), generated using the Aging, Dementia, and Traumatic Brain Injury (TBI) Study Atlas. **Method:** Metric – Rank correlation, linkage method – average by cluster tightness.

d) Volcano plot of differentially expressed genes (DEA) for the TRP gene family (n = 27) in human brain structures from an aging cohort study (structures n = 4: hippocampus [HIP], temporal neocortex [TCX], white matter of the forebrain [FWM], piriform cortex [PCX]; donors n = 98), generated using the Aging, Dementia, and Traumatic Brain Injury (TBI) Study Atlas.

e) TRP gene signatures in normal versus dementia brains. Volcano plot of TRP gene expression (n = 27) in brain cell types (n = 17) from healthy (n = 11,164,441 cells) and dementia (n = 1,052,021 cells) patients, generated using the CZ CELLxGENE Discover Platform. Each cell type is labeled with a distinct color, enabling clear differentiation between neuronal and non-neuronal populations (see legend S9c).
